# Early dynamic ultrasound for neonatal hip instability: implications for rural Australia

**DOI:** 10.1186/s12887-017-0830-z

**Published:** 2017-03-21

**Authors:** Susan L. Charlton, Adrian Schoo, Lucie Walters

**Affiliations:** Flinders Rural Health South Australia, Flinders University, Vivienne st, Mount Gambier, 5290 South Australia

**Keywords:** Screening, Neonatal instability of hip, Dynamic ultrasound, Developmental dysplasia of hip

## Abstract

**Background:**

Neonatal instability of the hip (NIH), where the femoral head can move away from the acetabulum, in the first weeks of life, is an important risk factor for developmental dysplasia of the hip (DDH). In rural areas in Australia, there is a recent trend to increased late diagnosis of DDH. Clinical screening of infant hips, a common practice in Australia, is experience dependent. Best practice early screening techniques are still debated with different techniques and timing used internationally. This systematic review examines early dynamic ultrasound (eDUS) screening for hip instability in the first 6 weeks after birth, and the early interventions informed by these findings and considers the findings for the context of rural Australia.

**Methods:**

The Cochrane Library, Medline, CINAHL and PEDro were searched for original research or systematic reviews, and clinical studies 1998 to 2015 involving dynamic ultrasound. Critical Appraisal Skills Programme tools were used to appraise the studies.

**Result:**

Nineteen studies were included. Early Dynamic Ultrasound (DUS) is consistently described as a reliable assessment of NIH. Early DUS is recommended for risk factors including geographical areas of high prevalence. Approaches to early intervention of hips with excessive movement are somewhat discipline-related and include: primary prevention (advice), secondary prevention (abduction supports), and conservative management (removable splints).

**Conclusions:**

In the context of increased prevalence of DDH in rural Australia, contemporary evidence suggests that introduction of early DUS could provide rural infants with more effective screening than clinical examination alone. Targeted early advice about posturing and simple removable supports to abduct infant hips could prevent some cases of DDH in rural Australia.

## Background

In order to avoid developmental dysplasia of the hip (DDH), the metaphyseal growth and ossification of the neonatal cartilaginous acetabulum must occur around *“a properly seated femoral head”* [1]. Neonatal instability of the hip (NIH), where the femoral head can move away from the acetabulum, in the first weeks of life is therefore an important risk factor for DDH [2]. DDH is the most common notifiable musculoskeletal birth defect in Australia with an incidence of 7/1000 births in 2007 in South Australia [3].

Despite serious long-term consequences for children with late diagnosis of DDH, best practice early screening techniques are debated with considerable variation in routine screening protocols internationally. This systematic review investigates early dynamic ultrasound screening (eDUS) methods for the detection of NIH, and how results can inform early interventions to potentially prevent either missed cases or delayed onset DDH.

Clinical screening of all neonatal hips is currently accepted as the most economic assessment of hips in many European countries, UK, USA, Canada and Australia, with infants considered at risk of DDH or with hips demonstrating subluxation then undergoing Graf ultrasound examination at 6 weeks of age. Graf ultrasound [4], a morphological assessment of the infant hip, measures the angle of the roof of the acetabulum (alpha angle) and the percentage cover of the femoral head. It classifies hips as: mature, immature, or dislocated. The wide range in the Graf measurements prior to 6 weeks makes it an unreliable screening tool in early infancy [5].

Clinical screening for DDH relies on detection of hip subluxation or dislocation soon after birth using Barlow or Ortolani methods and is experience-dependent with skilled, trained and experienced operators more reliably performing the procedure [6, 7]. As few as 31% of cases of DDH diagnosed on Graf ultrasound have a history of recognised risk factors or positive findings on clinical examination [8, 9]. Some hips reported as normal on clinical examination soon after birth, are later found to be dysplastic. This suggests the potential for false negative results on clinical screening, however there is also the potential that DDH develops in infants in the weeks to months after birth. Increased rates of late diagnosed dysplasia, after 3 months of age, were reported in Australia between 1988 and 2003 [10]. In rural areas in Australia, there is a recent trend to increased late diagnosis of DDH in comparison to urban regions [11]. We propose that current screening protocols do not accurately find and address increased instability in hips in the first 6 weeks of life.

Dynamic ultrasound hip examination was reported as early as 1988 [12]. The two techniques assessing hip mobility are lateral dynamic ultrasound (LDUS) [13] and anterior dynamic ultrasound (ADUS) [14]. LDUS is performed with the infant lying supine, hip flexed in neutral abduction, while a posterior force is exerted along the line of the femur. Movement of the femoral head away from the acetabulum can be assessed with a laterally placed ultrasound transducer relying on operator sensitivity [15]. LDUS can also afford a visual assessment of the change in percentage cover of the femoral head with pressure. ADUS is performed anteriorly in the groin during the Palmen-Barlow Manoeuvre. This enables the operator to quantify (in millimetres) the movement of the hip within the acetabulum and detect excessive movement not identified on clinical assessment alone [2, 15, 16].

The purpose of this study is to explore the role of early dynamic ultrasound (eDUS) in screening and early intervention of NIH with a view to reducing the frequency of DDH. The primary outcome of the study is to describe the efficacy of eDUS and the secondary outcome to describe early interventions instigated as a result of eDUS findings. These results are then considered for the specific context of rural Australia.

## Methods

The Cochrane Library, Medline, CINAHL and PEDro databases were searched for articles from 1998 to 2015 using the following search terms:

- Developmental dysplasia of the hip, or congenital hip dysplasia, or neonatal instability of hip, or hip laxity, or hip dislocation, or hip abduction, or hip development, or alpha angle, or acetabular cover
- AND dynamic ultrasound, or anterior dynamic ultrasound, or ultrasound screen$ or ultrasound scan$
- AND screening, detection, or management, or conservative management, or outcome.

Three levels of screening were used. Initially titles of the articles were reviewed. Abstracts were then reviewed. Included were original research or systematic reviews in English, and clinical studies involving dynamic ultrasound. Excluded were expert opinion, case studies, and studies where the first ultrasound was not performed in the first 6 weeks after birth. Full articles were obtained for studies meeting the criteria above, and level 3 screening performed where inclusion required that eDUS studies dealt with at least one of the following categories of information: screening, initial management, conservative management or treatment outcomes. The search was broadened to include articles cited by this initial yield that met the study inclusion criteria.

Articles were appraised using Critical Appraisal Skills Programme (CASP) tools for evaluating randomised controlled studies, qualitative and quantitative research studies, based on the 1994 guides for medical literature. CASP is a robust tool developed by the Oxford Regional Health Authority from the educational methods of McMaster University of Canada [17], in response to the need to base health service decisions on sound evidence of clinical effectiveness. When the methodology was found to be valid and reliable, and the interpretation of results was appropriate, a more detailed appraisal was undertaken by SC using the remaining questions of the tool. A score was allocated for each item, giving a possible overall score of 10. Scores from 8 to 10 were considered high-level studies, from 4 to 7.5 reasonable studies and those with less than 4 of little value for this study. When SC had queries regarding the rigor of a study she discussed these with her supervisors (AS or LW) to develop a consensus view of the CASP score. Finally, the authors categorised early interventions occurring prior to 6 weeks for this group of hips into three groups: primary prevention, secondary prevention, and conservative management (Table 1).

**Table 1:** Early Interventions for DDH in first 6 weeks of life

• *Primary prevention*: advice and instructions on positioning to optimise hip flexion and abduction including avoiding swaddling with legs wrapped tightly together	
• *Secondary prevention*: double nappy techniques, removable splints and Frejka pillows	
• *Conservative management*: non-surgical splinting	

## Results

**Fig. 1:**
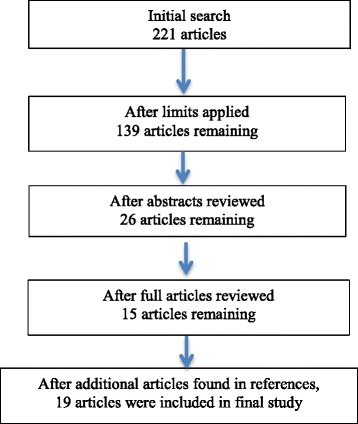
Flow chart of search results and studies included in the study

After applying the limitations, 139 articles were reviewed and of these, 26 articles were evaluated more comprehensively with 15 articles remaining and a further four added from citations in these papers (Figure 1).

Ten of the final articles were considered of high quality (8–10), eight of medium quality (4–7.5), and one scored low (Table 2). Five studies were systematic reviews [9, 18–21], two were prospective studies [2, 22], and the remaining studies were retrospective. Two were randomised controlled studies [16, 23]. Thirteen articles considered eDUS of “at risk” groups. Twelve papers studied universal eDUS.

**Table 2:** CASP evaluation of the different studies

Article	CASP Score	Detection		
		Clinical screening	Universal ultrasound	at risk’ ultrasound
Andersson [27]	9.5		y	
Bache [25]	9.5		y	
Holen [16]	9.5	y		y
Finnbogasan [2]	9	y		y
Finnbogasan [15]	9	y	y	y
Gomes [1]	9	y		y
Shorter [18]	9	y	y	y
Rosendahl [23]	9	y		
Shipman [9]	8	y	y	y
Elbourne [26]	8	y	y	
Clarke [24]	7.5	y		y
Rosendahl [20]	7.5		y	y
Chan [21]	7			y
Afaq [28]	6.5			y
Woolacott [19]	6.5	y	y	
Paton [22]	6.5	y		y
Harcke [29]	6		y	y
Kaisjer [30]	4	y	y	
Reikeras [31]	3	y		y

Excluding the literature reviews and two articles which made no comment on eDUS as a screening tool: four studies reported on LDUS [16, 24–26], six on ADUS [2, 8, 27–30] and two on both [1, 15] (Table 3). Again, excluding articles which reviewed the literature, ultrasounds were performed in the first week after birth in six studies [1, 16, 25, 27–29], the second week in two studies [2, 26], and in the first 8 weeks in two studies [26, 30]. Finnbogasan [15] examined ultrasound results from birth until 3 months.

**Table 3:** Early Dynamic Ultrasound study techniques

Name	US	Measurement in first 6/52	Timing	Follow-up	Stable	Unstable	Dislocatable	Dislocated	Measurement
Andersson [27]	ADUS	Yes	Week 1	4/52	<2 mm	2–4 mm	>4 mm	✔	0–4 mm
Finnbogasan [2]	ADUS	Yes	10–14 days		✔	✔	✔		Visual assessment
Afaq [28]	ADUS	Yes	Week 1	2/52 or 8/12	✔	✔	✔	✔	
Harcke [29]	ADUS	No	Week 1	Reg-8/52	✔	✔	✔	✔	Visual assessment
Gomes [1]	L & ADUS	No	Week 1	4/52 and 3/12	1–2 mm	2–4 mm	4–5 mm	5 mm	
Woolacott [19]	L & ADUS	Yes	Week 1	6/12 to 1 year	✔	✔	✔		Functional measure
Finnbogasan [15]	L & ADUS	No	Birth-3/12		✔	✔	✔	✔	Visual and measured
Paton [22]	L & ADUS	No	2/52 cl. Inst.	8/52 + risk fact	✔	✔	✔	✔	% cover α angle
Bache [25]	LDUS	Yes	Week 1	2–6/52	✔	✔	✔	✔	Visual and measured
Holen [16]	LDUS	Yes	Day 3	2/52	✔	✔	✔	✔	% cover α angle
Rosendahl [23]	LDUS	Yes	Day 4	6/52	≥60°	≤50°	≤43°	<43°	α angle
Elbourne [26]	LDUS	No	2–6/52	8/52	✔	✔	✔	✔	% cover α angle
Clarke [24]	LDUS	Yes	10/7 or 6/52	12/52	✔	✔	✔	✔	% cover α angle
Rosendahl [20]	LDUS	No	Week 1	Reg-8/52	✔	✔f/u 2/52	✔f/u 3/52	✔	
Chan [21]	LDUS	No	6/52		✔	✔	✔	✔	% cover α angle
Kaisjer [30]	ADUS	Yes	1 to 2 weeks	6/52 to 4/12	✔	✔	✔		measured
Reikeras [31]	LDUS	Yes	2–4/52	2 and 16 weeks	✔	✔	✔		measured
Shipman [9]	LDUS	No	Birth-6/12	2 years	✔	✔	✔		Functional measure
Shorter [18]	LDUS	Yes	Birth-4/12	1 year	✔	✔	✔		measured

NB ✔symbol indicated operator makes a qualitative assessment: either categorising to Graf levels, or simply a visual assessment

ADUS can be used reliably up to 6–8 weeks after birth [2, 15, 16]. After that time, the increasing strength and weight of the baby make accurate performance of the Palmen Barlow test difficult. The amount of movement is higher immediately after birth attributed to infant uptake of the hormone relaxin in late pregnancy [25]. Some authors have proposed normal ranges of movement seen on ADUS of hips over weeks 1–6 of life, ranging 1 mm–6 mm and progressively reducing with age [15, 27]. Andersson’s robust study positions his normal range findings (<2 mm) as most valid in contemporary western contexts [27].

Early studies in this review period recognised that eDUS studies improve the diagnostic value of static morphologic ultrasound studies [1, 26] particularly when performed by trained providers [21, 28]. Most studies recommended eDUS for infants with risk factors including: family history, breech position, foot deformity, and equivocal clinical assessment [2, 21, 25, 28]. One small study expressed concern about the operator dependence of eADUS results [30]. One study demonstrated that eADUS of infants with risk factors for DDH can lower the treatment rates [2]. However, clinical examination and eDUS identify overlapping groups of newborns, with some infants having solely positive clinical examination findings and others having solely eDUS evidence of instability [9]. So eDUS can result in additional hips being subjected to early intervention [18, 19]. At risk screening was recommended over universal screening because of: low prevalence, the natural history of NIH to commonly improve spontaneously, and the cost of eDUS [15, 21, 28]. As many infants diagnosed with DDH did not have traditional risk factors, a number of authors proposed eDUS screening for additional groups including: all girls, units where neonatal clinical screening was not of high quality, and in geographical areas with high prevalence [9, 16, 20, 21, 25]. Cost effectiveness of universal eDUS screening was recognised as dependent on national health system models (such as found in Scandinavia), however several studies recognised that this is changing with more litigious societies and lower cost scanning options being developed [24, 27, 29].

Timing and types of early intervention following eDUS are reported in Table 4, and results demonstrate attempts to tailor interventions in the first 3 months according to eDUS findings. In eight papers reporting early interventions the first authors are radiologists [1, 2, 13, 15, 20, 23, 26, 28], six are orthopaedic surgeons [16, 21, 22, 24, 25, 31], and two are paediatricians [27, 30].

**Table 4:** Intervention following early Dynamic Ultrasound

		Early intervention type and timing		Follow up
Name	Timing of eDUS	Unstable/ Subluxable	Dislocatable	
Radiologists/Radiographers				
Rosendahl [23]	Day 4	Surveillance	Frejka pillows or Surveillance	6 months
Elbourne [26]	2 weeks	Instability rescan 8 weeks	Splinting 2 weeks Secondary prevention	2 years
Finnbogasan [2]	10–14 days	Secondary prevention- Frejka pillow	Secondary prevention- Von Rosen	Not recorded
Afaq [28]	Week 1	Surveillance	Pavlik Harness 2 weeks Conservative management	8 months
Gomes [1]	Week 1	Surveillance	No treatment before 3/12	Not recorded
Harcke [29]	Week 1	Surveillance	Secondary prevention	Not recorded
Finnbogasan [15]	12 days	Surveillance	Secondary prevention	Not recorded
Rosendahl [20]	Week 1	Surveillance	Surveillance	6 months
Orthopaedic surgeons				
Bache [25]	Week 1	Re-examined 2 weeks	Pavlik harness for 6 weeks	12 months
Clarke [24]	10 days or 6 weeks	Conservative management -Pavlik harness 6 weeks	Pavlik harness / surgery	12 months
Paton [22]	2 weeks or 8 weeks	Secondary Prevention	Conservative management	Not recorded
Reikeras [31]	2–4 weeks	50% surveillance 50% primary prevention		12–14 months
Holen [16]	Day 3	US 2 weeks Frejka pillow 4 weeks	Secondary prevention	6–11 years
Chan [21]	6 weeks	Conservative management	Conservative management	Not recorded
Paediatricians				
Andersson [27]	Week 1	Primary Prevention R/V 2 weeks	Secondary prevention R/V 4 weeks	18 months
Kaijser [30]	Day 3	Secondary Prevention R/V 6 weeks	Conservative management R/V 12 weeks	Not recorded

There was consensus from the majority of papers in Table 4 that dislocated hips require immediate referral for either secondary prevention or conservative management using various splinting methods. Only two early papers proposed surveillance alone for dislocated hips in the first 8 weeks [1, 20]. There was also agreement that hips deemed to be stable on eDUS required no further intervention.

There were inconsistent approaches to early interventions for unstable/subluxable hips between studies. A number of studies recognise that eADUS screening provides opportunity for more frequent surveillance only [1, 15, 20, 23, 25, 26, 28, 29]. Many of these studies advocate delaying splintage until after 6 weeks of age to allow for spontaneous improvement [15, 25, 28, 29]. Only one paper, by a paediatrician mentioned primary prevention for unstable/subluxable hips [27]. This paper did not specifically discuss education of parents and carers about positioning and handling for optimal development of the infant hip [27]. Six studies considered secondary prevention in the first 6 weeks including: double nappy techniques, removable splints and Frejka pillows [2, 16, 22, 24, 30, 31]. Two studies described conservative management including non-surgical splinting with Pavlik harness [21, 24]. None of the studies in this literature review described baseline information about how infants with NIH were wrapped and positioned in the first months of life. Although it is commonly recognised that early intervention for DDH is frequently less invasive, only one small study (n = 41 hips with NIH Frejka pillows for 16 weeks cf n = 44 hips with NIH in control group) considered the rates of infant hips needing management after 8 weeks of age following secondary prevention [31]. Finally only one study considered conservative management (Pavlik harness) soon after initial ultrasound, and although this concluded that treatment rates were acceptable at 5–15 per 1000 births, it did not comment on avascular necrosis rates [24]. Of note is the discipline-specific preferences, with radiologists unlikely to consider early intervention other than surveillance, and orthopaedic surgeons greater focus on conservative management.

## Discussion

This literature review did not support universal eDUS screening of neonatal hips, however contemporary recommendations support eDUS for areas of high prevalence and reduced operator reliability of clinical screening. Rural Australia can be considered a geographical area with high prevalence of DDH where a concerning trend in increased late diagnosis has been found [10, 11]. Rural Australia is also a context where few babies are examined by paediatricians. Operator reliability of clinical screening using the Barlow and Ortolani tests requires constant practice, continued education and accurate follow up [32]. In Australian rural settings low birthing numbers may limit clinician experience and distance limits paediatric examination and feedback. We therefore propose eDUS could assist clinical judgement, improve quality of screening, and prevent missed diagnoses of NIH in rural Australia.

Austria and some other parts of Europe, including parts of Scandinavia, advise universal eDUS [33]. The US Task Force on hip recommendations prefers universal screening, although recognises this cannot be implemented due to ultrasound not being available everywhere in the US and due to a shortage of suitably trained sonographers. They therefore advise universal clinical assessment plus selective ultrasound for those infants with risk factors or equivocal clinical assessment [28]. Although both LDUS and ADUS have been used for early screening, Andersson’s robust quantitative values for normal hips and NIH suggest this method may be more consistently applied in rural Australia. A recent study in rural Australia has demonstrated the feasibility and acceptability of early ADUS screening [34]. Routine screening with eDUS may be more economically viable as portable ultrasound machines become more affordable and are further incorporated into contemporary clinical practice in rural Australia and internationally.

Based on the literature reviewed, there is little evidence to support conservative management with Pavik harness immediately after eDUS finding of NIH. Recently, Australia has seen increasing rates of late diagnosis of DDH (after 3 months) [10, 35]. The successful education program about prevention of Sudden Infant Death Syndrome, advocating placing babies in the supine position for sleeping, has led to many babies never being placed in prone in their early weeks. The reported increase in baby settling difficulties [36] and resurgence of swaddling to settle babies leads to increased extension and adduction of the infant hip [35]. Wrapping occurred simultaneously with a move away from bulky cloth nappies that better maintained infant hips in abduction and flexion. Secondary prevention, with double nappy techniques, removable splints and Frejka pillows, could theoretically reduce DDH in rural Australia. Although evidence for early secondary prevention in human studies remains thin, studies with animals show the effect of position on hip development is much more marked in the immediate weeks after birth [37]. Secondary prevention may be particularly attractive for parents in rural Australia where these interventions can be arranged locally, preventing referral and travel to distant specialist services.

Primary prevention measures in the first 6 weeks of life, including advice and instructions on positioning to optimise hip flexion and abduction, including avoiding swaddling with legs wrapped tightly together, could easily be instigated as routine practice in rural Australia. Certainly, an early educational program in Japan, highlighting the dangers of traditional swaddling, demonstrated improvement in hip development with significantly lower numbers of infants with late occurring DDH [38], showing that early education can enhance optimal infant hip development. The prone position, with hips flexed and abducted, promotes good hip development. Protective turning of the head, a primitive neonatal reflex seen in newborns, diminishes over the first few months and many babies are not happy on their tummies if they have not experienced the position during their first weeks [35]. Supervised awake tummy time should be encouraged from the earliest weeks. This literature review demonstrates a clear gap in the literature around how primary prevention strategies might impact on NIH. More research is required to understand the impact of identifying hips at risk with EDUS and providing targeted education on posturing in rural Australia.

The limitations of this article is translating best evidence to the context of rural Australia where sparse populations and distance significantly impact on access to specialist services. Strengths of this study include; that it distinguishes between hip laxity (NIH) and dysplasia (DDH), and the use of the CASP tools ensured a focus on studies of acceptable rigor.

### Conclusions

In the context of increased DDH in rural Australia, contemporary evidence suggests that introduction of eDUS could provide rural infants with more effective screening than clinical examination alone. Targeted early interventions such as improved postural management for optimal hip development, and simple removable supports to abduct infant hips could prevent some cases of DDH.

## Abbreviations

ADUS: Anterior dynamic ultrasound
CASP: Critical appraisal skills programme
DDH: Developmental dysplasia of the hip
DUS: Dynamic ultrasound
eDUS: Early dynamic ultrasound
LDUS: Lateral dynamic ultrasound
NIH: Neonatal instability of the hip
US: United States
